# Common drugs and treatments for cancer and age-related diseases: revitalizing answers to NCI's provocative questions

**DOI:** 10.18632/oncotarget.890

**Published:** 2012-12-30

**Authors:** Mikhail V. Blagosklonny

**Affiliations:** ^1^ Department of Cell Stress Biology, Roswell Park Cancer Institute, Elm and Carlton Streets, Buffalo, NY, USA

**Keywords:** cancer, aging, senescence, science

## Abstract

In 2011, The National Cancer Institute (NCI) has announced 24 provocative questions on cancer. Some of these questions have been already answered in “NCI's provocative questions on cancer: some answers to ignite discussion” (published in Oncotarget, 2011, 2: 1352.) The questions included “Why do many cancer cells die when suddenly deprived of a protein encoded by an oncogene?” “Can we extend patient survival by using approaches that keep tumors static?” “Why are some disseminated cancers cured by chemotherapy alone?” “Can we develop methods to rapidly test interventions for cancer treatment or prevention?” “Can we use our knowledge of aging to enhance prevention or treatment of cancer?” “What is the mechanism by which some drugs commonly and chronically used for other indications protect against cancer?” “How does obesity contribute to cancer risk?” I devoted a single subchapter to each the answer. As expected, the provocative questions were very diverse and numerous. Now I choose and combine, as a single problem, only three last questions, all related to common mechanisms and treatment of age-related diseases including obesity and cancer. Can we use common existing drugs for cancer prevention and treatment? Can we use some targeted “cancer-selective” agents for other diseases and … aging itself.

## INTRODUCTION

The National Cancer Institute (NCI) has announced 24 provocative questions on cancer for grant applications. Some of these really important questions could be answered at least in part in 2011, [1] just based on the existing knowledge, linking unrelated fields of science and medicine. Such an approach is very effective. For example, retrospective analysis of the effect of beta-blockers and metformin on breast cancer given for treatment hypertension and diabetes, respectively, has revealed their cancer-preventive effects in humans [2-6]. The questions answered in “NCI's provocative questions on cancer: some answers to ignite discussion” [1] were very diverse: from oncogene-addiction to curability of some disseminated tumors.

Here I choose to extend and update the answers to 3 related questions. Can we use our knowledge of aging to enhance prevention or treatment of cancer? What is the mechanism by which some drugs commonly and chronically used for other indications protect against cancer? The last question “How does obesity contribute to cancer risk?” is also related, since obesity is age-related disease (despite it can be easily caused or prevented by simple environment tactic – diet) and aging is a common mechanism for both aging and cancer as we have discussed [1]. Some common treatments emerged to prevent/suppress both obesity and cancer. Here I combine three related questions into a single problem, suggesting common treatments against some age-related diseases for cancer prevention as well as experimental anti-cancer agents for treatment of some age-related diseases and possibly aging.

### Can we use our deepening knowledge of aging to enhance prevention or treatment of cancer?

First of all, cancer is an age-related disease. The links between cancer and aging were discussed previously [1] and I will not be repeating them over here. Both senescent and cancer cells share the senescent phenotype [7, 8], including hyper-secretion [9-16]. The main difference between cancer and aging is that the control of cell cycle is disabled in cancer (by either loss of tumor suppressors that inhibit cell cycle (e.g., p16 [17-22]) or activations of activators of the cell cycle such as Myc [7, 23-39]. When the cancer cell is arrested by p16 induction, for instance, it becomes senescent (gerogenic conversion). But this is mTOR (and similar growth-promoting pathway) that drives gerogenic conversion (geroconversion) [7]. An arrested cancer cell is a senescent cell, whereas a proliferating senescent cell is a cancer cell [7]. Normal cells can become senescent by activation cytoplasmic nutrient-, mitogen-, stress-sensing and growth-promoting pathways such as mTOR. (I suggest the term gerogenic pathways, for brevity). If the cell cycle is blocked, such over-stimulated cells undergo gerogenic conversion (geroconversion), becoming senescent. What is common in cancer and senescence is the activation of growth-promoting signaling pathways such as mTOR [7, 40]. The mTOR pathway is constantly active in cancer cells due to mutations in receptor kinases, Ras, Akt, or loss of tumor suppressors (e.g., PTEN, TSC-2). [40-55]. Oncogenic transformation and gerogenic conversion are very close phenomena, involving similar signal-transduction molecules such as mTOR. Cancer and aging are not rivals but rather two faces of the same coin. In this mini-review, I will not discuss other very interesting aspects of the relationship between cancer and aging, as well as the meaning of aging because it have been discussed [56], [57], [58-60] and also because the involvement of gerogenic (oncogenic) pathways driving geroconversion (a conversion from cell cycle arrest to senescence [7], [8, 40]) is “the knowledge that can be used for cancer prevention”.

Geroconversion can be decelerated by rapamycin. A serious of experiments performed in diverse mammalian cells and models, demonstrated that mTOR is in fact involved in cellular aging [61-72], or strictly speaking, to gerogenic conversion. Furthermore, mTOR is involved in cell senescence and stem cell exhaustion in the organism [73-79].I need to repeat that rapamycin and other inhibitors of mTOR decelerate geroconversion [7]. But when geroconvesion is completed, rapamycin cannot reverse the event entirely. It is easier to decelerate and prevent senescence then reverse it. Eventually, hyperfunctions of the cells may be changed to malfunctions and the cells may become irresponsive to signals, including mTOR activators.

Calorie restriction (CR) deactivates the nutrient-sensing mTOR pathway and delays both aging and cancer (and other age-related diseases) [80-86]. Very importantly, short-term CR suppresses cellular senescence in the organism [87, 88].

Now it would not be surprising that, inhibition of mTOR decelerates organismal aging [60, 89 [109-113], and the [60, 108, 114]. Noteworthy, basal fasting levels of mTOR activity are increased in old mice [115]. Fasting is less effective in inhibiting mTOR, than rapamycin in mice [116].

Rapamycin and other rapalogs, metformin, as well as potential inhibitors of gerogenic pathways (currently under investigation in our laboratory) could be used for cancer prevention. But may other commonly used drugs can inhibit gerogenic pathways? This is a topic of the next question.

### PQ-5: Given the evidence that some drugs commonly and chronically used for other indications, such as an anti-inflammatory drug, can protect against cancer incidence and mortality, can we determine the mechanism by which any of these drugs work?

Certain drugs used for hypertension, atherosclerosis, diabetes, inflammation and immunossupression can protect against cancer. These drugs include rapamycin and other rapalogs, metformin, beta-blockers, angiotensin-blockers, aspirin. Since cancer is an age-related disease, drugs that inhibit gerogenic pathways may prevent cancer. At conventional doses, these accidental cancer-preventive agents are relatively ineffective to treat cancer, implying that their cancer-preventive effects are not due to targeting cancer cells directly. Aspirin: The anti-inflammatory agent aspirin, decreases inflammation, one of hallmarks of senescent cells. In some cell models, salicylate acid and aspirin inhibit the mTOR pathway [117, 118]. Aspirin decreases cancer incidence in humans [119-128]. Angiotensin-II-blockers. Inhibitors of angiotensin II activity include ACE inhibitors (such as captopril and lisinopril), which decrease angiotensin II production, and angiotensin receptor blockers such as losratan. Angiotensin-II-blockers suppress in hepatocarcinogenesis in rats [129] chemically-induced colon carcinogenesis obese mice and metastasis in mice [129-132]. In humans, use of these drugs is associated with a lower incidence of cancer occurrence [133-137]. In patients with renal transplantation, the use of angiotensin-II-blockers is associated with a two-fold reduced risk of skin cancers [138].

Angiotensin-II activates the mTOR pathway and causes cellular hypertrophy [139-149]. Therefore, angiotensin-II-blockers, which prevent these effects, are indirect inhibitors of mTOR.

Beta-blockers, which are used for therapy of hypertension, prevent breast cancer [2-4, 150-152]. There are several publications that activators of beta-androgenic receptors can activate the mTOR pathway [153, 154]. Therefore, beta-blockers are expected to block mTOR activation.

Rapamycin decelerates geroconversion (conversion of quiescence into senescence) in arrested cells [61-68, 155, 156]. Also rapamycin suppresses yeast aging and prolongs life span in *Drosophila* and mice [60, 89-113]. Rapamycin should delay cancer by slowing down the aging process. In fact, rapamycin prevents cancer in mice [157-164, 104, 105], and humans [165-169].

Finally, rapamycin prevents cancer in mice [104, 105, 112, 113, 157-164] and humans [165-169]. Its cancer-preventing effect may be indirect, due to prevention of senescent of normal stroma [170].

Metformin, an anti-diabetic drug, inhibits the mTOR pathway [171-173]. Metformin slows down aging, delays cancer and extend life span in rodents [174-182]. Also metformin decreases the risk of cancer in humans [5, 6, 183-193]. Metformin also exerts direct anti-cancer effects [54, 194-197]. Clinical studies in the neoadjuvant and adjuvant settings are ongoing; additional Phase 2 trials in the metastatic setting and proof of principle studies in the prevention setting are planned [198].

The NCI's questions “How does obesity contribute to cancer risk?” was discussed previously [1] and references within. Here I first outline the most important points discussed in [1]. There are many theories on how obesity promotes cancer, which mostly all are partially correct because there are simultaneously several mechanisms of how obesity contribute to cancer and each theory is based on some of them [199], [200], [201-205]

Without discussing them again [1], I emphasize one universal mechanism that obesity promotes cancer by over-activating the nutrient-sensing mTOR pathway in both normal and cancer cells.

Although obesity is an age-related disease, both genetic predisposition (other then age-related quasi-programmed genes) and especially environment play enormous roles. Obesity can be often induced independent of aging process by simple overeating. But still almost all people would gain weight after 30, unless they actively restrict their food intake. Most fitness-conscious people do, but unfortunately many others do not. As an aside, successful restriction of caloric intake can be considered by itself a treatment for obesity. Still visceral fat, the most dangerous for the human health, accumulates in old animals and humans, compared with younger animals and humans. Obesity is a disease that accelerates all other age-related diseases: diabetes, kidney disease, atherosclerosis, liver fibrosis, hypertension, the propensity to blood clots, neurodegeneration, sarcopenia, osteoporosis and of course cancer. Obesity accelerates aging and dramatically shorten life span. The links between obesity and cancer are direct, indirect and as well as causative and correlative. In all cases, mTOR is involved [1].

We can summarize the following mechanisms [1]:

a. Obesity can promote cancer directly by secretion several factors, including pro-inflammatory, by the adipose tissue and can directly stimulate tumor growth.
b. Obesity causes hormonal changes such as insulinemia and insulin promotes cancer.
c. Obesity can promote cancer by accelerating aging, obesity can accelerate aging and aging promotes cancer.
d. Aging can promote both obesity and cancer.
e. The relationships between them have been shown previously (in figure 2 [1]).

Also as we have already discussed [1], nutrients and insulin activate mTOR, whereas calorie restriction (fasting) deactivates mTOR. The mTOR pathway promotes obesity and is activated in obesity. Taking all together, one can conclude that rapamycin must prevent obesity.

In fact numerous studies demonstrated that rapamycin prevented obesity in mice on high fat diet. Yet, it was also shown that prevention of obesity may be associated with development of insulin-resistance or even diabetes-like condition, since chronic high-dose administration of rapamycin inhibits MTORC2 [206]. This stirred a controversy about rapamycin safety at chronic doses, especially in lay media. However, in depth analysis reveals that this condition resembles “starvation diabetes” described by Claude Bernard almost two centuries ago [207.] This condition was even observed during especially severe calorie restriction in humans and still was beneficial for their health [208]. As I already discussed, during starvation the organism needs to preserve glucose to feed the brain using as a tool insulin resistance in the liver, fat and muscle, lypolisis in the fat cells, glycogenesis and ketogenesis in the liver. Starvation diabetes is not a true type II diabetes [209]. I named it benevolent diabetes or type zero diabetes [209]. In fact, despite benevolent diabetes, mice live longer. In contrast, type II diabetes (true diabetes) promotes nephropathy, retinopathy, atherosclerosis and coronary disease. In contrast, rapamycin prevented these complications of true diabetes such as nephropathy and retinopathy [210, 211] Rapamycin prevents atherosclerosis in rodents [212, 213] and coronary re-stenosis in humans [214, 215]. Most importantly, a recent publication by Piguet et al supports the concept of benevolent diabetes: Rapamycin impacts positively on longevity, despite glucose intolerance induction [216].

There are many paradoxes related to insulin resistance and longevity (see for ref. [209]). All of them can be solved by classification of conditions in two groups: low mTOR versus high mTOR [209]. Calorie restriction and benevolent diabetes are beneficial, because they are associated with low mTOR. Rapamycin has many advantages compared with starvation: starvation may lead to malnutrition. (This may explain a well-publicized case, why calorie restriction did not extend life span of rhesus monkeys, despite decelerating aging [217]). In humans, rapamycin-induced diabetes is a rare complication even in transplant patients receiving high doses of rapamycin every day (see for ref. [209]).

But regardless of whether rapamycin-induced condition is benevolent, it can be avoided, until we know whether it contributes to lifespan extension or not (just an association). To avoid inhibition of TORC2 by rapamycin and insulin resistance, rapamycin should be used in intermittent fashion or in pulzes [209]. Preliminary data demonstrate that rapamycin tended to decreases insulin and obesity when given intermittently [116]. Intermittent rapamycin also prevents cancer [218, 112].

## CONCLUSIONS

Currently chemotherapy is the cornerstone of cancer treatment. It can cause cancer regression, remission and in rare cases even cure cancer. The arsenal of drugs and novel methods of their use, new strategies are constantly growing [219- 228] among many others but the progress in treatment is modestly incremental. Combining of anti-cancer drugs together increase their potency [90, 221, 229-234] but usually against both cancer and normal cells. Although in experiments performed in cell culture, chemotherapy can kill any cells, it is toxic to normal cells too (especially to hematopoietic and epithelial) and death from chemotherapy limits cancer therapy. There are several solutions. Exploiting some features of cancer cells, it is possible to protect selectively normal cells from chemotherapy without protecting cancer cells. This was demonstrated in paired cancer cells and normal cells in culture [235-251] and even in mice [252] but clinical trials have been never done. Another approach is to develop less toxic agents targeting cancer specific pathways (Ras, MEK, ERK, PI3K, IGF-1 and insulin, ErbB and other growth factors receptors, AMPK, mTOR, p70 S6 kinase, p53, oncogenic metabolism [] [228, 253-259]. The number of targeted approaches is rapidly growing [219-225] Such agents have been developed but most of them are too “weak”, not cytotoxic enough, and resistance rapidly develops [260, 261]. However, this Achilles’ heel of signal transduction inhibitors could be used for treatment two purposes: First, for protection of normal cells from chemotherapy especially when cancer cells are resistant [237, 262, 263]. Second, at low and intermittent doses for treatment of age-related diseases and aging itself. As you may noticed, many of the targets of such anticancer drugs (PI3K, IGF-1R, PDGFR HER2 and other growth factors receptors and signaling molecules such as AMPK, mTOR, p70 S6 kinase, ATM, p63, p53 are involved in aging and therefore, in all age-related diseases, one of which is cancer. Therefore, we can envision administration of such inhibitors at low, intermittent, non-toxic doses, which are not intended to damage cells. To prevent cancer by gerosupression, they need to be administered not at high doses but in low, intermittent doses. Alternatively, they could be used as protectors of normal cells from chemotherapy (rapamycin and nutlin are examples) [241-251].

On the other hand, thousands of drugs have been developed for treatment of age-related diseases (obesity, hypertension, atherosclerosis, cardiac arrhythmias an so on) either semi-empirically or by intentional development of inhibitors of prostaglandin synthesis, beta-receptor, growth-factor receptors, angiotensin II, testosterone signal-transduction pathways. All these drugs are inhibitors of signal transduction pathways involved in diseases. Many of them, for example angiotensin II, inhibit “hyperfunctional” cells by activating mTOR. Many of these conventional drugs are already known to inhibit the mTOR pathway. It does not seem to be a coincidence that drugs that treat age-related diseases inhibit gerogenic pathways (given that aging itself is caused by hyperactivation of signal transduction pathways such as mTOR). Therefore, it would not be surprising to expect that some of conventional drugs used in the clinic would have cancer preventive activities, since cancer is an age-related disease. Therefore retrospective studies of some commonly used drugs for cancer preventive effects are warranted. The most trivial and at the same time are amazing examples (by its genius simplicity) is calorie restriction or fasting. Calorie restriction and fasting both slow down aging. They are recommended for almost all age-related diseases (except for terminal conditions). Calorie restriction and intermittent fasting prevent or delay cancer. Recently, it was shown that short term complete fasting decreased the side effects of chemotherapy in cancer patients [246, 247, 264, 265]. Since calorie restriction and fasting are not so efficient as rapamycin in inhibition of mTOR and also may cause malnutrition, rapamycin at low and/or intermittent doses may be even a better choice for prevention of side effects of chemotherapy.
